# Stable Gold@Polydopamine@ssDNA Bioconjugates for Highly Efficient Detection of Tumor-Related mRNA in Living Cells

**DOI:** 10.3390/molecules30173551

**Published:** 2025-08-29

**Authors:** Senhao Hu, Wenjing Wang, Yu Zou, Chunmei Li, Hongyan Zou, Chengzhi Huang, Lei Zhan

**Affiliations:** Key Laboratory of Biomedical Analytics, Chongqing Science and Technology Bureau, College of Pharmaceutical Sciences, Southwest University, Chongqing 400715, China

**Keywords:** Au@PDA bioconjugates, tumor-related mRNA, Ca^2+^-assisted DNA adsorption, multiplex imaging

## Abstract

The development of low-background, facile, and robust fluorescent nanoprobes for imaging and monitoring of intracellular mRNA changes remains a great challenge. Taking advantage of the high fluorescence quenching efficiency of core-shell gold@polydopamine (Au@PDA) nanocomposites and Ca^2+^-promoting DNA adsorption stability, a simple and universal bioconjugate strategy was designed to a construct fluorescent nanoprobe for highly efficient tumor-related mRNA imaging. The fluorescence of Cy5-labeled DNA was quenched up to 92.38% by the AuNP and PDA via nanometal surface energy transfer (NSET) and photoinduced electron transfer (PET), respectively. TK1 mRNA, a biomarker of tumor growth, initiates hybridization and results in fluorescence recovery, which built the foundation for identifying the expression level changes in living cells. More importantly, three kinds of tumor-related mRNA (TK1 mRNA, GalNAc-T mRNA, and C-myc mRNA) can be detected simultaneously with different fluorophore-modified recognition sequences, which can avoid false positive signals and improve the reliability of cancer diagnostic, holding great promise for cancer diagnosis, prognosis, and therapy.

## 1. Introduction

Tumor-related messenger RNAs (mRNAs), such as TK1 mRNA, MUC1 mRNA, GalNAC-T mRNA, C-myc mRNA, and survivin mRNA, have been suggested as specific biomarkers for tumor progression and prognosis [1]. The identification of mRNA type and accurate expression level determination hold great promise for tumor diagnosis and categorization, cancer staging or grading, and therapeutic effect monitoring [2]. Taking thymidine kinase 1 (TK1) as an example, elevated TK1 expression is an early event in tumorigenesis and correlated with poor prognosis in various types of cancers. More recently, it has been shown that high expression of TK1 is significantly associated with immune suppression during cancer development [3]. In addition, the TK1 expression level is essential for cellular sensitivity to trifluridine (FTD), the active component of chemotherapeutic drug FTD/TPI, which is approved to treat refractory metastatic colorectal cancer [4]. Therefore, identifying and tracking intracellular tumor-related mRNAs is important in early diagnosis and prognostic assessment of tumors.

DNA-conjugated gold nanoparticle (AuNP)-based nanomaterial surface energy transfer (NSET), a technique capable of measuring distances up to 40 nm in comparison with normal fluorescence resonance energy transfer (FRET), has emerged as a reliable tool for the real-time, in situ detection of intracellular-specific RNA [5,6]. These fluorescent DNA-AuNP probes are usually composed of a reporter sequence and recognition sequence on the AuNP surface and detect intracellular mRNA by the release of a fluorescently labeled reporter sequence [7,8,9]. Despite the impressive properties of these probes, the utility is generally limited by the complex modification process [10], low energy transfer efficiency [11], and high false-positive rate in biological fluids [12]. It remains a challenge to construct facile, robust, and universal fluorescent probes for long-term intracellular detection of mRNAs.

Polydopamine (PDA), a versatile agent for surface modification, has attracted considerable interest since it was reported in 2007 [13]. Benefiting from its ease of synthesis, excellent biocompatibility, unique optical properties, and strong adhesive properties, numerous PDA-coated hybrid materials have been reported and applied successfully in biomedical researches such as tissue engineering, drug delivery, photothermal therapy, and biosensing/bioimaging [14]. Among these, PDA-modified gold nanoparticles can effectively enhance detection sensitivity due to their high stability and strong interference resistance, holding significant promise for biomedical applications [15,16]. Notably, PDA can act as an electron acceptor to quench fluorescence with high efficiency because of photo-induced electron transfer (PET) [17,18]. In addition, single-stranded DNA (ssDNA) has been found to be adsorbed on PDA by π–π stacking, hydrogen bonding, or Lewis acid–base interactions. It should be noted that polyvalent metal ions such as Ca^2+^, Zn^2+^, Mn^2+^, and Co^2+^ may help DNA adsorption, which enables high stability of the DNA-AuNP probe to avoid displacement by competing ligands in biological systems effectively [19,20].

**Figure 1:**
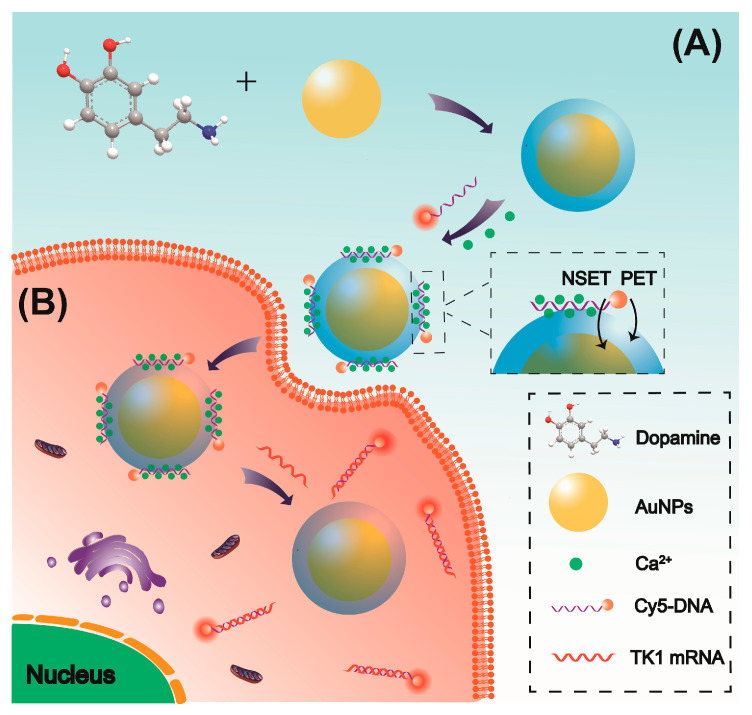
Schematic representation of the Au@PDA bioconjugates designed for the determination of tumor-related mRNA based on NSET and PET strategy. (**A**) Preparation of stable Au@PDA bioconjugates with Ca^2+^-mediated DNA immobilization. (**B**) Principle of mRNA-induced fluorescence turn-on for Au@PDA bioconjugates in living cell.

In this work, a simple and stable nanoprobe was constructed for long-term imaging of intracellular TK1 mRNA in living cells. Au@PDA core–shell nanoparticles were created by in situ polymerization of dopamine onto the surface of AuNP under alkaline conditions (Figure 1). Cy5-modified ssDNA can be assembled on the surface of Au@PDA with strong affinity, and the adsorption was further promoted by Ca^2+^ via metal coordination. Subsequently, the fluorescence of Cy5 can be quenched effectively by PDA and AuNPs based on PET and NSET mechanisms. In the presence of TK1 mRNA, specific binding between the Cy5-labeled ssDNA probe and TK1 mRNA triggers the release of the reporter probe from the PDA shell, and a detectable fluorescence signal was generated subsequently, which enabled quantitative studies of TK1 mRNA with low background interference. Moreover, the Au@PDA conjugates allow one to simultaneously visualize the expressions of multiple tumor-related mRNAs in living cells, improving the accuracy of early diagnosis of cancer.

## 2. Results and Discussion

### 2.1. Characterization of AuNP and Au@PDA Bioconjugates

**Figure 2:**
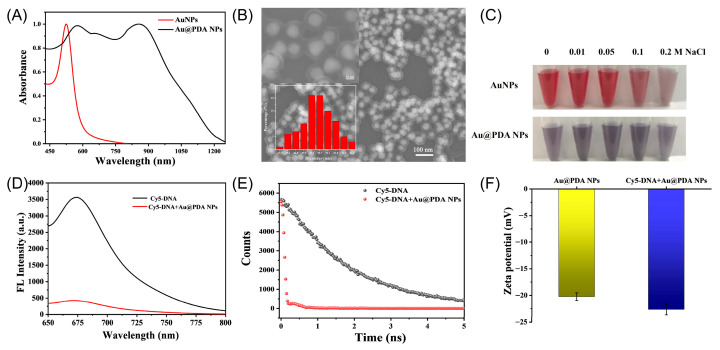
Characterization of AuNPs and Au@PDA NPs. (**A**) UV–vis absorption spectra of aqueous AuNPs and Au@PDA NP solutions. (**B**) Representative SEM image of the synthesized Au@PDA NPs (insets: high-definition SEM image and size distribution). (**C**) Stability of AuNPs and Au@PDA NP dispersions with different concentrations of NaCl. (**D**,**E**) Fluorescence spectra and time-resolved PL decay of Cy5-ssDNA in presence of or absence of Au@PDA NPs. (**F**) Zeta potential of Au@PDA NPs alone and Au@PDA NPs with Cy5-ssDNA functionalization in H_2_O.

Citrate-protected AuNPs were prepared following a seeded growth strategy via the reduction of HAuCl_4_ with sodium citrate. The optical property of the AuNP solution measured by UV–vis spectroscopy is depicted in Figure 2A. It shows a characteristic surface plasmon resonance (SPR) band with maximum absorbance at 526 nm. The SEM result indicated that as-prepared AuNPs are nearly spherical and narrow in size with an average diameter of 38 nm (Figure S1). The hydrodynamic diameter of AuNPs is around 56 nm, as determined by DLS, demonstrating good monodispersity in H_2_O. Meanwhile, AuNPs showed a negative Zeta potential due to the citrate coating.

Au@PDA NPs were easily obtained by dispersing AuNPs in dopamine solution. Compared to the AuNPs, Au@PDA NPs displayed a broader SPR peak and a solution color of purplish grey, which was contributed by the PDA layer formation. The core–shell structure consisted of a core of AuNPs and a shell of PDA, where an average diameter of 50 nm was clearly observed via SEM (Figure 2B). It is noted that the thickness of the outer PDA shell could be easily adjusted by changing the amount of dopamine. Four different Au@PDA NPs (Au@PDA-1, Au@PDA-2, Au@PDA-3, and Au@PDA-4) were obtained with the same AuNP core but different dopamine concentrations at 0.05 mg/mL, 0.1 mg/mL, 0.2 mg/mL, and 0.4 mg/mL, respectively. As the dopamine concentration increased, the thickness of the PDA layer increased as expected, accompanied by an enlargement in the overall particle size of the Au@PDA (Figure S2). Correspondingly, the hydrodynamic diameter of the Au@PDA NPs increased from 148.4 nm to 373.3 nm (Figure S3), which is consistent with the SEM results. Although the hydrodynamic diameter of the same Au@PDA nanoparticles is larger than the SEM-measured size, this discrepancy arises because the two techniques characterize different aspects of the particles. SEM is typically used to confirm the actual morphology of the particles, whereas DLS is employed to assess their size and stability in relevant application environments (Table S1). As well, PDA is a kind of ampholyte polymer containing catechol and amine functional groups, and it is generally negatively charged in neutral/basic conditions. The Zeta potential results also showed that all Au@PDA NPs had a negative surface charge (Figure S4), attributed to the deprotonation of phenolic groups on the PDA shells.

The stability of AuNPs and Au@PDA NPs in high-ionic-strength solutions was further investigated (Figure 2C). The solution of AuNPs showed a significant color change after adding 0.2 M NaCl, while the Au@PDA NP solution had no obvious change. The UV spectra further demonstrated that Au NP absorbance intensified with higher NaCl concentrations, in contrast to Au@PDA NPs, which exhibited negligible change in absorbance (Figure S5). These results demonstrate the high stability of Au@PDA NPs in high-ionic-strength solutions. In addition, there were no discernible color and structural changes for over 4 weeks, and their integrity and dispersity were also maintained in aqueous solution.

### 2.2. Interaction Between DNA Probe and Au@PDA Bioconjugates

The fluorescence quenching performance of the Au@PDA NPs toward the dye-labeled single-strand DNA probe was investigated (Figure 2D). A strong fluorescence emission at 670 nm was observed in the Cy5-attached ssDNA. However, with the introduction of Au@PDA NPs, the fluorescence was almost completely quenched, indicating the high fluorescence quenching efficiency of the Au@PDA NPs. Corresponding to the fluorescence changes, the average fluorescence lifetime of Cy5 probes decreased from 1.90 to 0.14 ns, which fit the biexponential decay (Figure 2E), suggesting that the dyes may be deposited on the surface of the internal pores [21]. Since PDA contains rich catechol groups, which provide the negatively charged surface, it hindered the adsorption of ssDNA. However, the π–π stacking and hydrogen bonding between the nucleobases of ssDNA and aromatic/amino groups of PDA may be contributed to the facile and direct loading of ssDNA [22]. The Zeta potential with ssDNA attachment was more negative than that of Au@PDA NPs alone (Figure 2F), suggesting the successful adsorption of Cy5-DNA onto the surface of Au@PDA NPs. Notably, with the coordination of Ca^2+^, ssDNA was found to be adsorbed on PDA effectively, which was quantified with a NanoDrop spectrophotometer (Figure S6). Furthermore, the stability of the Au@PDA-ssDNA probe with the assistance of Ca^2+^ was assessed through a BSA displacement experiment (Figure S7). The results showed that Cy5-DNA adsorbed on Au@PDA NPs with Ca^2+^ was more stable in the presence of BSA.

### 2.3. Coexistence of Charge and Energy Transfer Process

PDA, with abundant semiquinone and quinone functional ligands, has been widely employed as an excellent electron transfer acceptor to construct fluorescent assays for biomolecules [18,23]. Specifically, experimental and theoretical evidence from previous studies showed that electrons can transfer from the photoexcited dyes to the lowest unoccupied molecular orbital (LUMO) of the proximal quinone structure of PDA [24,25]. In addition, the NSET process between Cy5 and AuNPs in this work, where AuNPs served as the energy acceptor and Cy5 as the energy donor, also produced substantial quenching of Cy5 fluorescence because the emission spectra of Cy5 overlapped with the AuNPs’ absorption spectra.

To offer deeper insights into the quenching mechanism, charge and energy transfer processes between Cy5 and Au@PDA pairs have been addressed (Table S2). Firstly, the total efficiency ($E_{total}$) of the quenching process was related to the fluorescence lifetime of the excited-state donor and can be obtained from the following expression:

(1) $$E_{total}=1-\frac{\tau_{DA}}{\tau_{D}}$$

where $\tau_{DA}$ and $\tau_{D}$ are the fluorescence lifetime of the Cy5-ssDNA with or without Au@PDA NPs, respectively. The total decay rate (*k*_total_) of the Au@PDA-Cy5 complex is equal to the sum of all potential deactivation rates:

(2) $$k_{total}=k_{r}+k_{nr}+k_{NSET}+k_{PET}=\frac{1}{\tau_{DA}}$$

where $k_{r}$ and $k_{nr}$ are the radiative decay rate and the nonradiative decay rate, respectively. $k_{NSET}$ is the NSET rate, and $k_{PET}$ is the PET rate.

(3) $$k_{0}=k_{r}+k_{nr}=\frac{1}{\tau_{D}}$$

The efficiency of NSET (*E*_NSET_) is proportional to the fourth power of the distance between donor and acceptor. Therefore, the estimated NSET efficiency was obtained using the expression:

(4) $$E_{NSET}=\frac{{\left(R_{0}^{NSET}\right)}^{4}}{{\left(R_{0}^{NSET}\right)}^{4}+R^{4}}=\frac{k_{NSET}}{k_{total}}$$

where $R_{0}^{NSET}$ is the distance when the quenching efficiency between the donor and acceptor is 50%, while R is the actual distance from donor to acceptor surface. The specific calculation formula of $R_{0}^{NSET}$ is as follows:

(5) $$R_{0}^{NSET}={\left(0.225c^{3}Q_{D}W_{D}^{2}W_{F}^{-1}K_{F}^{-1}\right)}^{\frac{1}{4}}$$

where c is the speed of light, $Q_{D}$ and $W_{D}$ represent the quantum yield of the donor and the angular frequency of donor emission, respectively, and $W_{F}$ and $K_{F}$ are the angular frequency and the Fermi vector for bulk gold, respectively. The PET rate ($k_{PET}$) was estimated from the expression:

(6) $$k_{PET}=k_{total}-k_{NSET}-k_{0}$$

Hence, the PET efficiency ($E_{PET}$) was calculated:

(7) $$E_{PET}=\frac{k_{PET}}{k_{total}}$$

The calculated *E*_total_ was 92.38% according to the fluorescence lifetime of the donor, and $R_{0}^{NSET}$ was 6.79 nm. For calculating NSET efficiency, the thickness of PDA was 11.3 nm, which was directly used as the actual distance from the donor to the acceptor surface by ignoring the distance between Cy5 and PDA. Therefore, $E_{NSET}$ and $E_{PET}$ were 12.86% and 79.52%, respectively.

### 2.4. Optimization of the Experimental Conditions

To obtain the best performance for TK1 mRNA detection, the shell thickness of PDA, Cy5-ssDNA concentration, reaction temperature, and incubation time were systematically analyzed. To evaluate the influence of the PDA shell thickness on the performance of the probe for detecting TK1 mRNA, four distinct types of Au@PDA NPs (Au@PDA-1, Au@PDA-2, Au@PDA-3, and Au@PDA-4) were tested in terms of their fluorescence recovery rate in the presence of the target and Cy5-ssDNA fluorescence quenching efficiency. The results indicated that the fluorescence quenching efficiency of Au@PDA NPs toward Cy5-ssDNA increased with greater shell thickness. However, the highest fluorescence recovery efficiency was achieved with Au@PDA-3, which was consequently selected and applied for subsequent TK1 mRNA detection experiments (Figure S8). Subsequently, the amount of the Cy5-ssDNA probe was optimized, since the Cy5-ssDNA bonded to the surface of Au@PDA with high affinity, affecting the fluorescence response significantly (Figure S9A). The quenching efficiency of Au@PDA gradually increased with the Cy5-ssDNA concentration ranging from 150 to 450 nM, but excess Cy5-ssDNA could generate obvious background signals. Meanwhile, the signal-to-background (*F*/*F*_0_) ratio, where *F* and *F*_0_ represented, respectively, the fluorescence intensities with and without target mRNA, reached the maximum when the concentration of Cy5-ssDNA was 450 nM. Taken together, 450 nM Cy5-ssDNA was adopted as the optimal amount for mRNA detection. In addition, the effect of the reaction temperature and time on detection was tested. The fluorescence recovery resulting from the 37 °C incubation was significantly higher than that from 25 °C at the same concentration of the target mRNA (Figure S9B). And the fluorescence signal increased in the range from 15 to 60 min and reached a plateau beyond 60 min, indicating that target mRNA had been fully hybridized with Cy5-ssDNA adsorbed on Au@PDA NPs (Figure S9C). Thus, the best experimental conditions for TK1 mRNA detection were incubation at 37 °C for 60 min. Finally, the optimal concentration of the Au@PDA NPs with the target mRNA was 1.64 nM, and the highest *F*/*F*_0_ was observed (Figure S9D).

### 2.5. Analytical Performance of AuNP@PDA-Based Method

**Figure 3:**
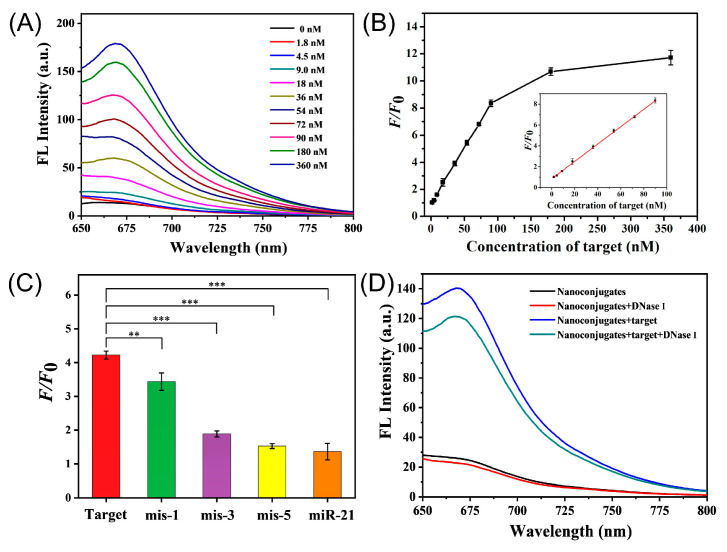
TK1 mRNA detection using Au@PDA NPs as probe. (**A**) Fluorescence spectra of nanoconjugate in response to different concentrations of TK1 mRNA. (**B**) Relationship between the fluorescence ratio (*F*/*F*_0_) and the TK1 mRNA concentration. Inset: linear relationship. (**C**) Fluorescence response of nanoconjugate with TK1 mRNA, mismatched TK1 mRNA, and miR-21. (**D**) Fluorescence spectra after hybridization of the nanoconjugate with TK1 mRNA in the presence or absence of DNase I. Data are presented as the mean ± SD (*n* = 3); ** *p* < 0.01, and *** *p* < 0.001.

The performance of AuNP@PDA-based method for the detection of TK1 mRNA was evaluated under the optimal conditions. As indicated in Figure 3A, increased fluorescence signal recovery was obtained for various concentrations of TK1 mRNA addition. The changes in *F*/*F*_0_ were found to be proportional to the concentration of the target mRNA in the range of 1.8 to 90 nM, with a calibration equation of *F*/*F*_0_ = 0.0829 × *c*_target_ + 0.8647 (*R*^2^ = 0.9991) (Figure 3B). The limit of detection (LOD) was calculated to be 0.43 nM based on the 3σ rule. Although there was still a gap compared to the electrochemical method for TK1 mRNA detection, this strategy possessed a comparable or superior linear range and detection sensitivity compared with the other previously reported fluorescence methods (Table S3). Furthermore, the specificity of Au@PDA bioconjugates was investigated by treatment with base mutation strands and another interfering miRNA. Notably, one-base-mismatched (mis-1), three-base mismatched (mis-3), and five-base mismatched (mis-5) strands could also be distinguished in the same condition, and nearly no signal recovery was observed for miR-21, indicating the high specificity of this method (Figure 3C).

### 2.6. Intracellular Operation of the Au@PDA-ssDNA

The stability toward enzymes and biocompatibility of the Au@PDA-ssDNA are very crucial for biological studies. The resistance of Au@PDA-ssDNA toward DNase I digestion was first examined in this work. The result demonstrated that there was only a slight difference in the fluorescence change of the reporter strand on the nanoconjugates upon incubation with DNase I (Figure 3D). And a significant difference in fluorescence was observed that was coming from the hybridization with the target but not the digestion of probe, indicating the apparent stability of Au@PDA-ssDNA nanoconjugates. Additionally, cell viability studies for Au@PDA-ssDNA were subsequently performed against LO2, HepG2, and MCF-7 cells using the CCK-8 assay (Figure S10). Although all cells showed slightly enhanced toxicity at a higher concentration of conjugates, no apparent change in cellular viability after 24 h of incubation was observed in the range from 0.25 to 1.0 nM (survival rate > 90%), showing the prospect of biological imaging.

**Figure 4:**
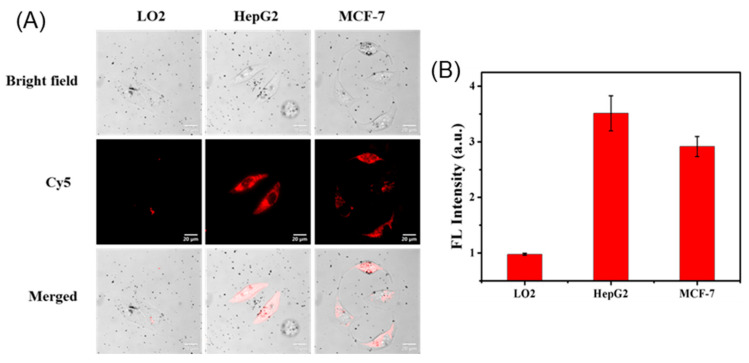
Intracellular imaging of TK1 mRNA using Au@PDA-ssDNA bioconjugates. (**A**) Confocal microscopy images in LO2, HepG2, and MCF-7 cells. (**B**) Quantitative analysis of fluorescence intensity in (**A**). Data are presented as mean ± SD (*n* = 5).

Having demonstrated good biocompatibility and stability, we next applied the Au@PDA-ssDNA conjugates to mRNA imaging in living cells. Different cell lines, HepG2, MCF-7, and LO2, were chosen because the TK1 mRNA expression levels in these cell lines are different [26]. As shown in Figure 4, Au@PDA-ssDNA conjugates entered the three cell lines effectively, as described in the reported works, benefiting from the negative surface charge [27,28]. Fluorescence images of high expression levels of TK1 mRNA were determined in the HepG2 and MCF-7 cells, whereas LO2 cells had low expression, reflecting the intrinsic variability in gene expression, which was connected to its biofunctional activity. Notably, as the Au@PDA-ssDNA concentration increased from 0.1 to 1.6 nM, three cell lines showed relatively strong fluorescence after incubation at a concentration of 0.8 nM (Figure S11). Besides the probe concentration, a positive correlation between cellular fluorescence intensity and incubation time was found in HepG2 cells. It was suggested that the 8 h incubation time should be suitable for imaging TK1 mRNA in the subsequent experiments (Figure S12).

To evaluate whether the fluorescent signal was truly generated by hybridization with the target mRNA, the Cy5-labeled ssDNA probe was replaced with polyA21-Cy5 to anchor on the Au@PDA NPs for cellular imaging. The majority of cells exhibited an extremely bright fluorescent signal throughout the cytoplasm with the Au@PDA-ssDNA probe, while cells incubated with Au@PDA-polyA21 produced a faint fluorescent signal, suggesting that Au@PDA-ssDNA conjugates can be applied for the specific imaging of endogenous mRNA in living cells (Figure S13).

**Figure 5:**
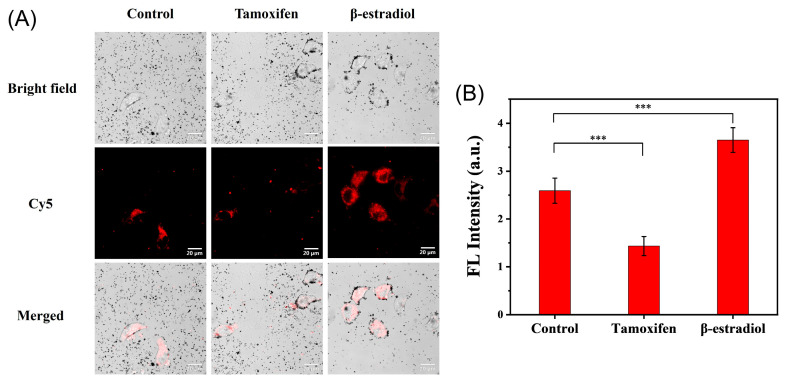
The specificity of TK1 mRNA analysis in HepG2 cells using bioconjugates. (**A**) Confocal microscopy images of HepG2 cells treated with β-estradiol and tamoxifen, respectively. (**B**) The average fluorescence intensity in (**A**). Data are presented as mean ± SD (*n* = 5), *** *p* < 0.001.

The expression of TK1 mRNA appears to be different at various stages of tumor progression and prognosis [29]. Monitoring the expression level of TK1 mRNA in living cells is of great significant for cancer detection and therapy. As previously reported, β-estradiol and tamoxifen could be generally used to regulate expression of tumor-related mRNA in cancer cells [30]. When HepG2 cells were treated with tamoxifen in this work, the average measurement of total cellular fluorescence intensity was significantly lower than that of the untreated cells, while the fluorescence intensity of cells treated with β-estradiol was stronger than that in control group (Figure 5). These results were consistent with other works, which found that tamoxifen and β-estradiol caused a reduction and enhancement in the global expression of TK1 mRNA, respectively [31,32].

### 2.7. Generality Evaluation of the Bioconjugates

To evaluate the feasibility for multiplex mRNA detection, we first evaluated whether the quenching ability for other fluorescent dye (FAM and Cy3)-labeled ssDNA was maintained. In the absence of Au@PDA NPs, free FAM-ssDNA and Cy3-ssDNA showed bright fluorescence emission at 530 nm and 563 nm, respectively. The introduction of Au@PDA NPs caused them to bind with ssDNA strongly to form bioconjugates with the aid of Ca^2+^ and quench the fluorescence of FAM and Cy3 efficiently (Figure S14). Furthermore, Au@PDA-FAM and Au@PDA-Cy3 with recognition sequences were designed and constructed to respond C-myc mRNA and GalNAc-T mRNA, wherein C-myc is a key regulator in promoting and maintaining tumorigenesis, and GalNAc-T is a key glycosyltransferase overexpressed in various types of human cancers [33]. Fluorescence signals generated from bioconjugates in the presence of the target mRNA increased with an increasing concentration of C-myc and GalNAc-T from 0 to 90 nM, suggesting the generality of this sensing platform based on Au@PDA NPs (Figure S15).

**Figure 6:**
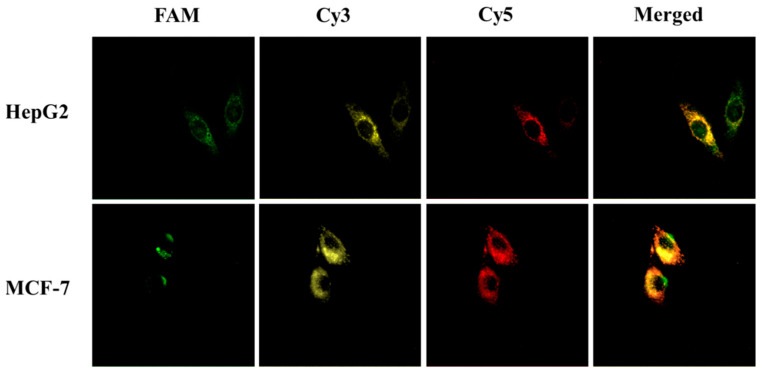
Intracellular fluorescence imaging of C-myc mRNA (FAM, excitation at 488 nm), GalNAc-T mRNA (Cy3, excitation at 543 nm), and TK1 mRNA (Cy5, excitation at 633 nm) using the bioconjugates.

Considering all mRNAs are associated with tumor progression, HepG2 and MCF-7 cells were selected to assess whether three mRNAs could be imaged simultaneously in living cells through Au@PDA-ssDNA conjugates. As shown in Figure 6, when HepG2 and MCF-7 were treated with the three bioconjugates, a strong green fluorescence signal for C-myc mRNA, a yellow fluorescence signal for GalNAc-T mRNA, and a red fluorescence signal for TK1 mRNA were presented via confocal fluorescence microscopy images, demonstrating that Au@PDA NPs hold promise as a general platform in multicolor imaging, which can improve the accuracy of cancer diagnosis.

## 3. Experimental Section

### 3.1. Materials and Reagents

All oligonucleotides were synthesized and purified by Sangon Biotechnology Co., Ltd. (Shanghai, China). The sequences of oligonucleotides used in this work are listed in Table S4. Chloroauric acid (HAuCl_4_·4H_2_O) was supplied by Sigma-Aldrich Chemical Co. (St. Louis, MO, USA). Trisodium citrate was purchased from Chengdu Kelon Chemical Reagent Factory (Chengdu, China). Dopamine hydrochloride was supplied by Aladdin Reagent Co., Ltd. (Shanghai, China). Tamoxifen and β-estradiol were purchased from Macklin Biochemical Co., Ltd. (Shanghai, China).

DMEM, RPMI 1640 medium, 0.25% (*w*/*v*) trypsin protease solution, and 1× phosphate-buffered saline (PBS) were purchased from GE Healthcare Life Sciences Inc. (Marlborough, MA, USA). Fetal bovine serum (FBS, Gibco) was supplied by Thermo Fisher Scientific Inc. (Waltham, MA, USA). Commercial cell counting kit-8 (CCK-8) was obtained from Dojindo Laboratories (Kumamoto, Japan).

### 3.2. Apparatus

Scanning electron microscopy (SEM) images were taken on a Hitachi S-4800 electron microscope (Tokyo, Japan) under an accelerating voltage of 30 kV. UV–vis absorption spectra were recorded with a Hitachi UV-3010 spectrophotometer (Tokyo, Japan). All fluorescence spectra were collected with a Hitachi F-4600 fluorescence spectrophotometer (Tokyo, Japan). The hydrodynamic diameter and ζ-potential of the as-prepared nanoparticles were determined on a Malvern Zetasizer Nano ZS. All fluorescence images were acquired by an Olympus IX2-DSU confocal scanning system with an Olympus IX-81 inverted microscope equipped with a Rolera-MGi EMCCD camera (Tokyo, Japan). All fluorescence lifetimes were recorded using the Fluorescence Spectrometer FLS1000 (Edinburgh, Livingston, UK) equipped with an NKT Supercontinuum laser (excitation wavelength: 640 nm; emission wavelength: 670 nm; repetition rate: 40 MHz; pulse period: 25 ns).

### 3.3. Preparation of AuNPs and Au@PDA NPs

AuNPs were synthesized according to a previously reported method [34]. Briefly, an aqueous solution of sodium citrate (150 mL, 2.2 mM) was added to a 250 mL round-bottomed flask equipped with a condenser and heated under magnetic stirring. After boiling, 1 mL of HAuCl_4_ (25 mM) was rapidly injected, and the mixture was then kept at 100 °C for 10 min with stirring. The yellow solution gradually turned soft pink, which indicated the formation of 10 nm-sized Au seeds. Then, the reaction solution was cooled to 90 °C and remained at this temperature for 5 min. Sodium citrate (60 mM, 1 mL) and HAuCl_4_ (25 mM, 1 mL) were added sequentially with an interval of 2 min. After continuous stirring for 30 min, additional sodium citrate (60 mM, 1 mL) and HAuCl_4_ (25 mM, 1 mL) were injected and reacted. This process was repeated five times. The resulting AuNP solution was then aged and cooled down to room temperature.

AuNPs were coated with a PDA shell by an ultrasonic-assisted self-polymerization of dopamine approach [35]. In brief, 10 mL of the dopamine solution prepared in Tris-HCl buffer (10 mM, pH 8.5) was injected into 10 mL of freshly prepared AuNP solution under continuous sonication for 1 h at room temperature. The Au@PDA NPs were separated by repeated centrifugation at 8000 rpm for 5 min and finally redispersed in water.

### 3.4. Au@PDA Bioconjugate Preparation

Au@PDA-based nanoprobes were created via metal ion-promoted DNA adsorption. In a typical experiment for TK1 mRNA detection, a 10 μM Cy5-labeled ssDNA probe (67 μL) was added to Au@PDA conjugates in HEPES buffer (10 mM, pH 7.6) with Ca^2+^ (2 mM). The mixture was subsequently incubated at room temperature for 1 h. Free reporter DNAs were removed by centrifugation with 8000 rpm for 5 min, and the Au@PDA-ssDNA conjugates were resuspended and stored at 4 °C for subsequent experiments. Other Au@PDA bioconjugates used in this work were prepared following the same procedure as described above.

To evaluate nuclease stability of the Au@PDA-ssDNA bioconjugates, DNase I (2 U/mL) was incubated with the nanoprobe at 37 °C for 1 h. The fluorescence spectra were recorded from 650 to 800 nm with excitation at 640 nm.

### 3.5. Cell Culture and Cytotoxicity Assay

MCF-7, HepG2, and LO2 cells were grown in DMEM or RPMI1640 medium supplemented with 10% FBS and 1% antibiotics (penicillin/streptomycin, 100 U/mL). The cells were maintained in a humidified incubator containing a 95% air and 5% CO_2_ atmosphere at 37 °C. The cell culture medium was replaced every other day. For the cytotoxicity assay, cells were separately cultured with different concentrations of bioconjugates for 24 h in 96-well plates and gently rinsed with sterilized PBS buffer three times. CCK-8 was used to assess the cell viability according to the manufacturer’s instructions.

### 3.6. mRNA Determination

Complementary mRNA determination in tubes was performed by mixing various concentrations of mRNA with Au@PDA bioconjugates in HEPES buffer solution. Taking TK1 mRNA as an example, 50 μL of the target with different concentrations (0, 1.8, 4.6, 9.0, 18, 36, 72, 90, 180, and 360 nM) was added to a 1.5 mL centrifuge tube containing the Au@PDA probe, and the mixture was diluted to 400 μL with water. After incubation for 1 h at 37 °C, the fluorescent signals were obtained under 640 nm excitation. For GalNAc-T mRNA and C-myc mRNA detection, Cy3 and FAM were excited at 514 and 660 nm, and their emission spectra were recorded from 550 to 640 nm and 500 to 600 nm, respectively.

To measure the selectivity of the Au@PDA-Cy5 conjugates, mismatched sequences were spiked into the detection solution under the same conditions, and the fluorescence was measured after the reaction. Each sample was independently prepared and measured three times for statistical analysis.

### 3.7. Imaging of mRNA in Living Cells

For imaging experiments, cells (MCF-7, HepG2, or LO2) were seeded into a glass-bottom dish at a density of 1 × 10^5^ cells/well and cultured overnight. The cells were washed three times with PBS and then incubated with Au@PDA bioconjugates in fresh medium containing 2% FBS at 37 °C for a required time. After culture, the dishes were washed, cells withdrawn from the culture medium, and washed briefly with PBS to remove any bioconjugates. Fluorescence images were acquired with an Olympus IX2-DSU live-cell confocal microscope (Tokyo, Japan). The resulting raw data and the relative fluorescence intensity were analyzed by ImageJ 1.54p and Image-Pro Plus v6 software, respectively.

For analyzing the expression level of TK1 mRNA, HepG2 cells were preincubated, respectively, with inhibitor (Tamoxifen, 1 μM) or stimulator (β-estradiol, 1 nM) for 24 h at 37 °C. After the treatment, cells were treated with the Au@PDA bioconjugates in culture medium for 2 h, and then a plate-based fluorescence assay was performed to analyze intracellular delivery.

For multiplex mRNA imaging, HepG2 and MCF-7 cells were seeded and grown until 80% confluency, followed by washing with PBS. A mixture of Au@PDA modified with each DNA probe, which was specific for the target mRNAs C-myc, GalNAc-T, and TK1-labeled FAM, Cy3, and Cy5 was added to the cells and incubated at 37 °C, followed by examination with a confocal microscope under different laser beams.

## 4. Conclusions

In summary, we successfully developed a robust, stable Au@PDA-ssDNA probe for detecting and imaging TK1 mRNA in living cells through the integration of the FRET and PET processes. Dye-labeled ssDNA was strongly adsorbed on the Au@PDA NP surface with Ca^2+^ coordination-mediated conjugation and transfected into the cytoplasm directly, achieving high specificity and sensitivity of the target mRNA. The probe demonstrates exceptional analytical performance for TK1 mRNA quantification, featuring a linear dynamic range of 1.8 to 90 nM (*R*^2^ = 0.9991) with a 0.43 nM detection limit. Remarkably, we have shown the capability of this nanoprobe in the discrimination of cells with different TK1 mRNA expressions and multiplexed detection of two other tumor-related mRNAs, allowing for accurate measurement in living cells. Given the reliability and generality of this strategy, Au@PDA-ssDNA may be readily extended to sense a wide range of intracellular biomarkers, so the probe holds significant promise for in vivo tumor profiling.

## Data Availability

The original contributions presented in this study are included in the article. Further inquiries can be directed to the corresponding author(s).

## Supplementary Materials

The following supporting information can be downloaded at: https://www.mdpi.com/article/10.3390/molecules30173551/s1, Figure S1: Scanning electron microscopy image of AuNPs obtained by seeded growth strategy. (insets: size distribution); Figure S2: SEM images of Au@PDA conjugates prepared with different concentration of dopamine (insets: size distribution): (A) 0.05 mg/mL, (B) 0.1 mg/mL, (C) 0.2 mg/mL and (D) 0.4 mg/mL; Figure S3: Hydrodynamic Size Distribution of AuNPs and Au@PDA conjugates prepared with different concentration of dopamine. Figure S4: Zeta Potential of AuNPs and Au@PDA conjugates prepared with different concentration of dopamine. Figure S5: UV absorption spectra of (A) AuNPs and (B) Au@PDA NPs dispersions in different concentration of NaCl. Figure S6: The amount of Cy5-DNA present in the supernatant after DNA was adsorbed by Au@PDA NPs and Au@PDA NPs/Ca^2+^. Figure S7: The fluorescence recovery ability of Cy5-DNA adsorbed on Au@PDA NPs and Au@PDA NPs/Ca^2+^ in the presence of BSA. Figure S8: (A) Quenching efficiency of four types of Au@PDA NPs to Cy5. (B) The effect of four types of Au@PDA NPs on the fluorescence recovery in the presence of target. Figure S9: Optimization of the experimental parameters for target mRNA detection. (A) The influence of the Cy5-ssDNA concentration on the fluorescencen recovery of Au@PDA-ssDNA. (B-D) Effect of (B) reaction temperature, (C) incubation time, and (D) calcium salts on the F/F0 value of the Au@PDA-ssDNA probe in the presence of 45 nM target mRNA. Figure S10: Cell viability of LO2, HepG2 and MCF-7 cells after incubation with different concentrations of nanoprobe. Figure S11: Confocal fluorescence micrographs for TK1 mRNA imaging with increased nanoprobe concentration (0, 0.1, 0.2, 0.4, 0.8, 1.6 nM) in cells. Figure S12: Confocal fluorescence micrographs for TK1 mRNA imaging in different incubation time (2, 4, 6, 8, 10, 12 h) in HepG2 cells. Figure S13: Fluorescent confocal micrographs of TK1 mRNA imaging with polyA21-Cy5 nanoprobe and nanoprobe in HepG2 cells. Figure S14: The changes of fluorescence intensity of the three dyes (FAM, Cy3 and Cy5) in the presence of Au@PDA NPs with different excitation wavelengths. (A) FAM labeled ssDNA (FAM-DNA) with 488 nm excitation wavelength. (B) Cy3labeled ssDNA (Cy3-DNA) with 540 nm excitation wavelength. Figure S15: Representative fluorescence spectra for nanoprobe in the presence of various concentrations of mRNA targets measured with different excitation wavelengths, respectively. (A) FAM labeled ssDNA (FAM-DNA) for C-myc mRNA detection with 488 nm excitation wavelength. (B) Cy3 labeled ssDNA (Cy3-DNA) for GalNAc-T mRNA detection with 540 nm excitation wavelength. Table S1: Hydrodynamic diameter and number-average diameter of AuNPs and Au@PDA conjugates prepared with different concentration of dopamine.; Table S2: Related parameters and results of calculating NSET and PET efficiency between Au@PDA NPs and Cy5. Table S3: The comparison of TK1 mRNA detection between this work and other fluorescent methods. Table S4: DNA sequences used in the experiment. References [36,37,38,39,40,41,42,43,44,45,46,47] are cited in the supplementary materials.
