# Supplementary material for: Stable Gold@Polydopamine@ssDNA Bioconjugates for Highly Efficient Detection of Tumor-Related mRNA in Living Cells
Source: Molecules. 2025 Aug 29;30(17):3551. doi: 10.3390/molecules30173551 (PMC12430423; doi:10.3390/molecules30173551)
Supplement: Supplementary file 1 [file molecules-30-03551-s001.zip › molecules-3774991-supplementary.pdf]

Supporting Material

for

Stable Gold@Polydopamine@ssDNA Bioconjugates for Highly Efficient Detection of Tumor-Related mRNA in Living Cells

Senhao Hu, Wenjing Wang, Yu Zou, Chunmei Li, Hongyan Zou*, Chengzhi Huang and Lei Zhan*

Key Laboratory of Biomedical Analytics (Southwest University), Chongqing Science and Technology Bureau, College of Pharmaceutical Sciences, Southwest University, Chongqing 400715, P. R. China.

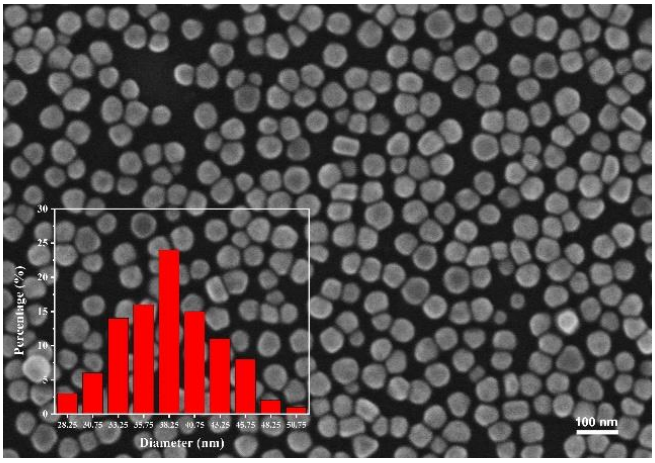

Figure S1 Scanning electron microscopy image of AuNPs obtained by seeded growth strategy. (insets: size distribution.)

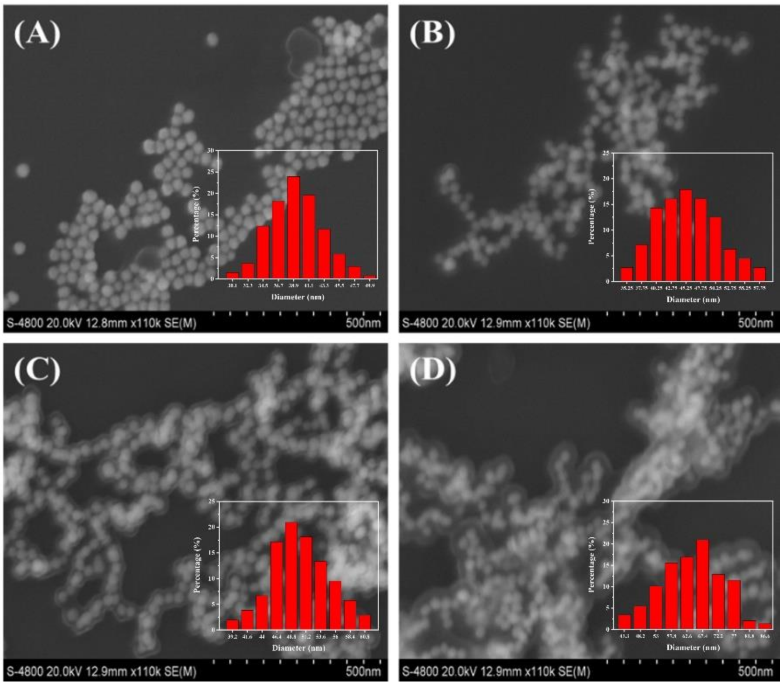

Figure S2 SEM images of Au@PDA conjugates prepared with different concentration of dopamine (insets: size distribution.): (A) 0.05 mg/mL, (B) 0.1 mg/mL, (C) 0.2 mg/mL and (D) 0.4 mg/mL.

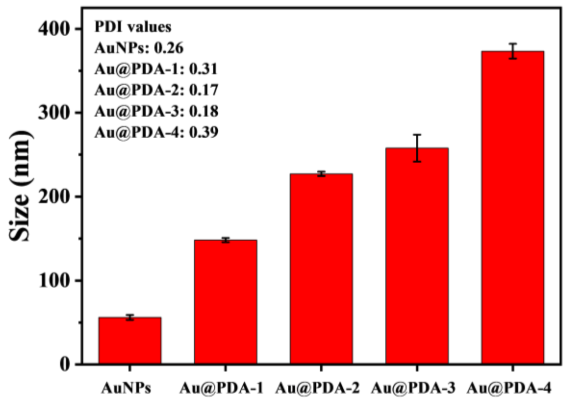

Figure S3 Hydrodynamic Size Distribution of AuNPs and Au@PDA conjugates prepared with different concentration of dopamine.

Table S1 Hydrodynamic diameter and number-average diameter of AuNPs and Au@PDA conjugates prepared with different concentration of dopamine.

Samples	Hydrodynamic Diameter (nm)	Number-Average Diameter (nm)
AuNPs	56	38
Au@PDA-1	148	39
Au@PDA-2	227	46
Au@PDA-3	258	50
Au@PDA-4	373	64

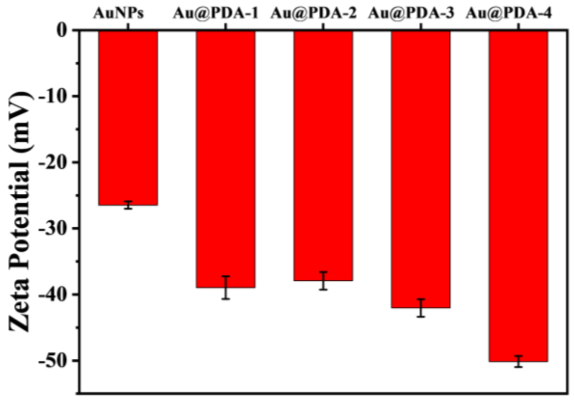

Figure S4 Zeta Potential of AuNPs and Au@PDA conjugates prepared with different concentration of dopamine.

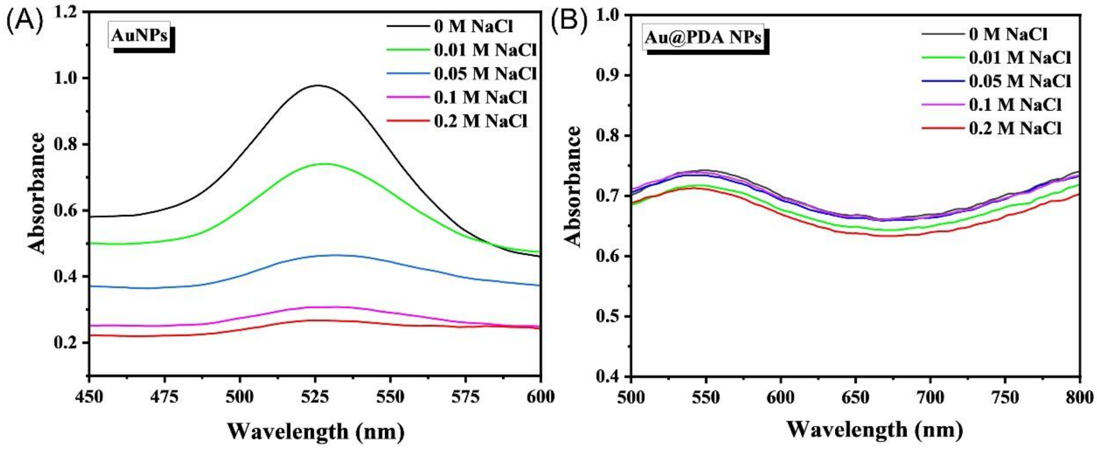

Figure S5 UV absorption spectra of (A) AuNPs and (B) Au@PDA NPs dispersions in different concentration of NaCl.

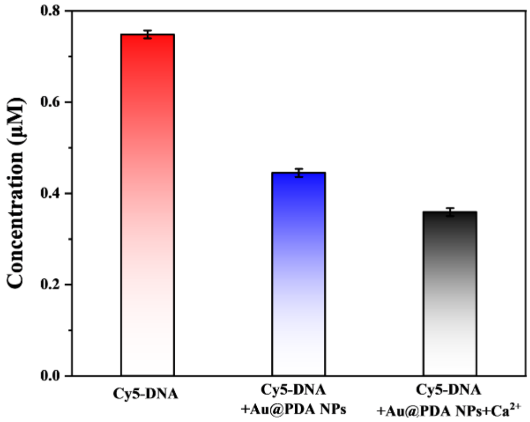

Figure S6 The amount of Cy5-DNA present in the supernatant after DNA was adsorbed by Au@PDA NPs and Au@PDA NPs/Ca^{2+}.

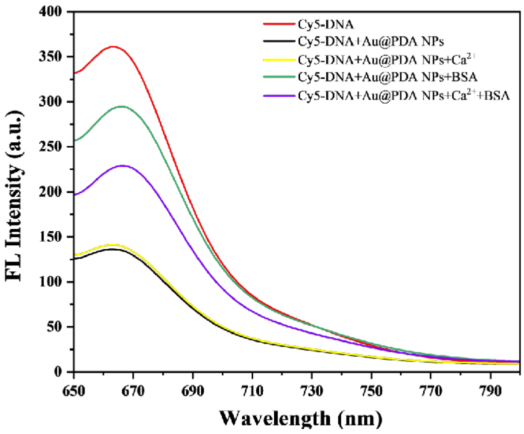

Figure S7 The fluorescence recovery ability of Cy5-DNA adsorbed on Au@PDA NPs and Au@PDA NPs/Ca^{2+} in the presence of BSA.

Table S2 Related parameters and results of calculating NSET and PET efficiency between Au@PDA NPs and Cy5.

Parameter	Value	Reference
c (m/s)	3×10^{8}	
Q_D	0.28	[1, 2]
W_D (S^{-1})	2.82×10^{15}	[3]
W_F (S^{-1})	8.4×10^{15}	[4]
K_F (m^{-1})	1.2×10^{10}	
E_{total} (%)	92.38	
R_0^{NSET} (nm)	6.79	
R (nm)	11.3	
E_{NSET} (%)	12.86	
E_{PET} (%)	79.52	

[1] S. Buckhout-White, C. M. Spillmann, W. R. Algar, A. Khachatrian, J. S. Melinger, E. R. Goldman, M. G. Ancona and I. L. Medintz, Nat. Commun., 2014, 5, 5615.

[2] W. P. Klein, S. A. Díaz, S. Buckhout-White, J. S. Melinger, P. D. Cunningham, E. R. Goldman, M. G. Ancona, W. Kuang and I. L. Medintz, Adv. Optical Mater., 2018, 6, 1700679.

[3] H. Y. Zou, P. F. Gao, M. X. Gao and C. Z. Huang, The Analyst, 2015, 140, 4121-41219.

[4] C. Chen, C. Midelet, S. Bhuckory, N. Hildebrandt and M. H. V. Werts, J. Phys. Chem. C, 2018, 122, 17566-17574.

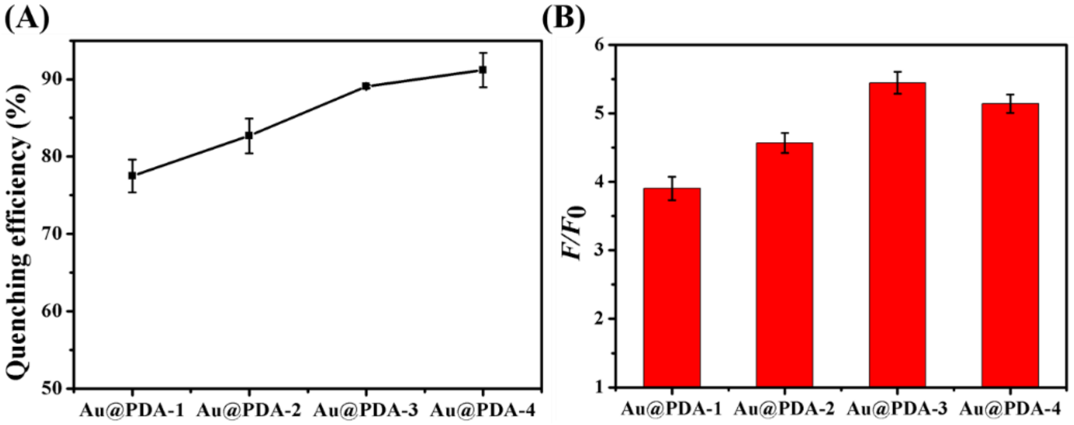

Figure S8 (A) Quenching efficiency of four types of Au@PDA NPs to Cy5. (B) The effect of four types of Au@PDA NPs on the fluorescence recovery in the presence of target.

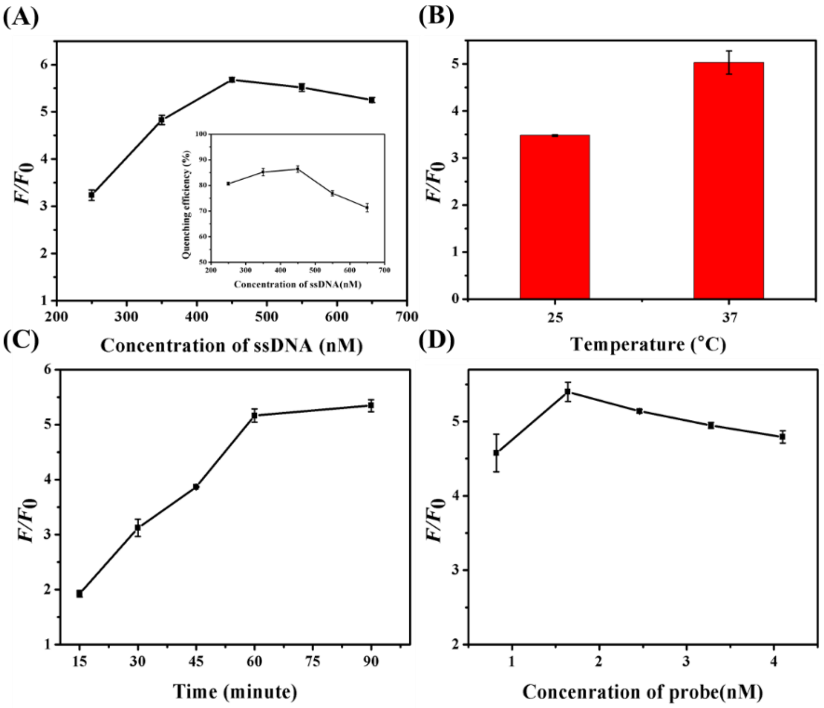

Figure S9 Optimization of the experimental parameters for target mRNA detection. (A) The influence of the Cy5-ssDNA concentration on the fluorescencen recovery of Au@PDA-ssDNA. (B-D) Effect of (B) reaction temperature, (C) incubation time, and (D) calcium salts on the F/F_0 value of the Au@PDA-ssDNA probe in the presence of 45 nM target mRNA.

Table S3 The comparison of TK1 mRNA detection between this work and other fluorescent methods.

Methods	Linear range (nM)	LOD (nM)	Ref.
Electrochemical	0.1–1.0	0.06	1
Electrochemical	0.1–10	0.022	2
Fluorescence	0–25	1.5	3
Fluorescence	5-80	1.34	4
Fluorescence	10–400	2.57	5
Fluorescence	1–20	0.7	6
Fluorescence	25–500	8	7
Fluorescence	10–50	0.2	8
Fluorescence	0-200	1.74	9
Fluorescence	1.8–90	0.43	This work

1. Dai, S.; Zhou, Y.; Dai, P.; Cheng, G.; He, P. and Fang, Y., The Split Primer Ligation-triggered 8-17 DNAzyme Assisted Cascade Rolling Circle Amplification for High Specific Detection of Liver Cancer-involved mRNAs: TK1 and c-myc. *Electroanalysis* **2020**, 32(3), 554-560.
2. Dai, S.; Zhou, Y.; Cheng, G.; He, P. and Fang, Y., Dual-signal electrochemical sensor for detection of cancer cells by the split primer ligation-triggered catalyzed hairpin assembly. *Talanta* **2020**, 217, 121079.
3. He, M.; He, M.; Nie, C.; Yi, J.; Zhang, J.; Chen, T., et al., mRNA-Activated Multifunctional DNAzyme Nanotweezer for Intracellular mRNA Sensing and Gene Therapy. *ACS Applied Materials & Interfaces* **2021**, 13(7), 8015-8025.
4. Li, T.; Sun, M.; Xia, S.; Huang, T.; Li, R.-T.; Li, C., et al., A binary system based DNA tetrahedron and fluorogenic RNA aptamers for highly specific and label-free mRNA imaging in living cells. *Talanta* **2024**, 269.
5. Jiang, Y.; Xu, X.; Fang, X.; Cai, S.; Wang, M.; Xing, C., et al., Self-Assembled mRNA-Responsive DNA Nanosphere for Bioimaging and Cancer Therapy in Drug-Resistant Cells. *Analytical Chemistry* **2020**, 92(17), 11779-11785.
6. Li, X.; Zou, R.; Chen, F.; Chen, C.; Gong, H. and Cai, C., Stimulus-responsive strategy based on MnO2 nanosheet-modified mesoporous silica nanoprobes for accurate multiple mRNAs detection. *Talanta* **2023**, 255, 124179.
7. Wu, M.-J. and Tseng, W.-L., Rapid, facile, reagentless, and room-temperature conjugation of monolayer MoS2 nanosheets with dual-fluorophore-labeled flares as nanoprobes for ratiometric sensing of TK1 mRNA in living cells. *Journal of Materials Chemistry B* **2020**, 8(8), 1692-1698.
8. Ma, W.; Chen, B.; Jia, R.; Sun, H.; Huang, J.; Cheng, H., et al., In Situ Hand-in-Hand DNA Tile Assembly: A pH-Driven and Aptamer-Targeted DNA Nanostructure for TK1 mRNA Visualization and Synergetic Killing of Cancer Cells. *Analytical Chemistry* **2021**, 93(30), 10511-10518.
9. Gong, H.; Yao, S.; Zhao, X.; Chen, F.; Chen, C. and Cai, C., Construction of an autofluorescence interference-free phosphorescence biosensor for the specific detection of TK1 mRNA. *Analytica Chimica Acta* **2024**, 1303.

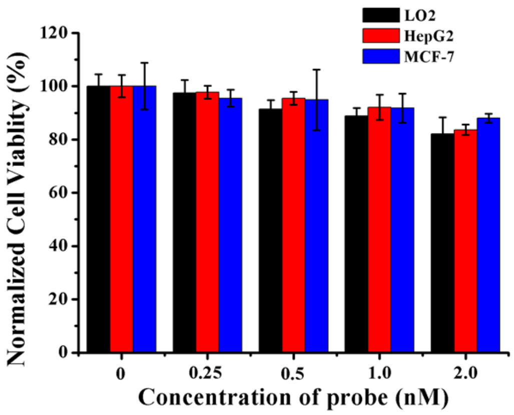

Figure S10 Cell viability of LO2, HepG2 and MCF-7 cells after incubation with different concentrations of nanoprobe.

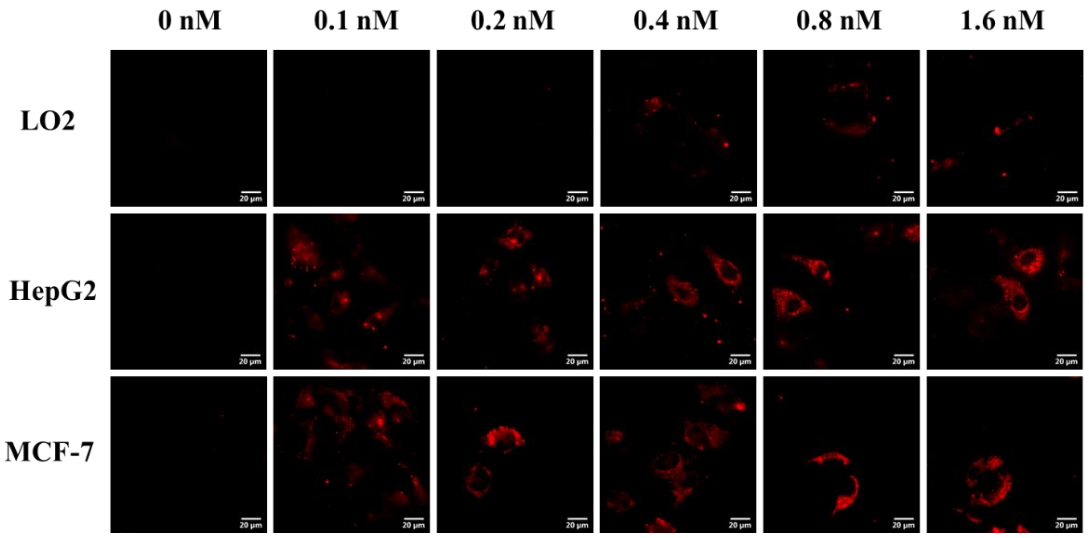

Figure S11 Confocal fluorescence micrographs for TK1 mRNA imaging with increased nanoprobe concentration (0, 0.1, 0.2, 0.4, 0.8, 1.6 nM) in cells.

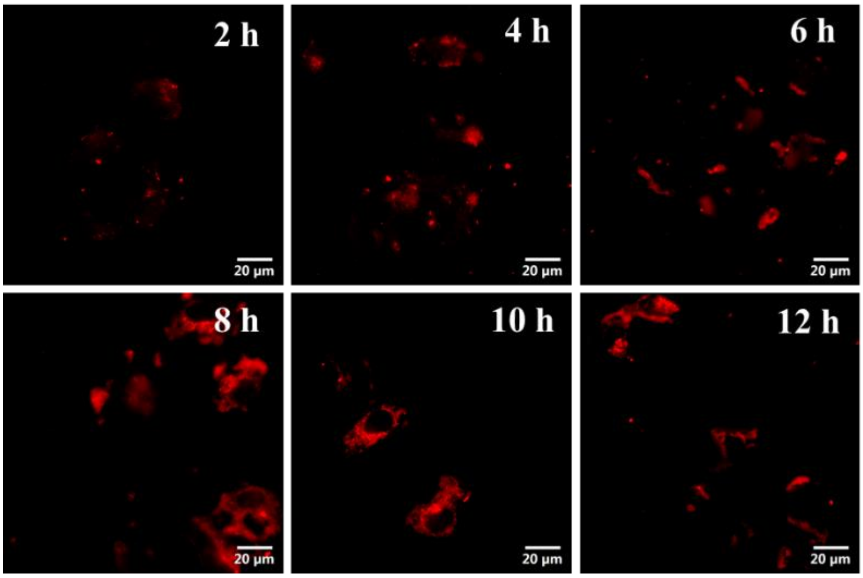

Figure S12 Confocal fluorescence micrographs for TK1 mRNA imaging in different incubation time (2, 4, 6, 8, 10, 12h) in HepG2 cells.

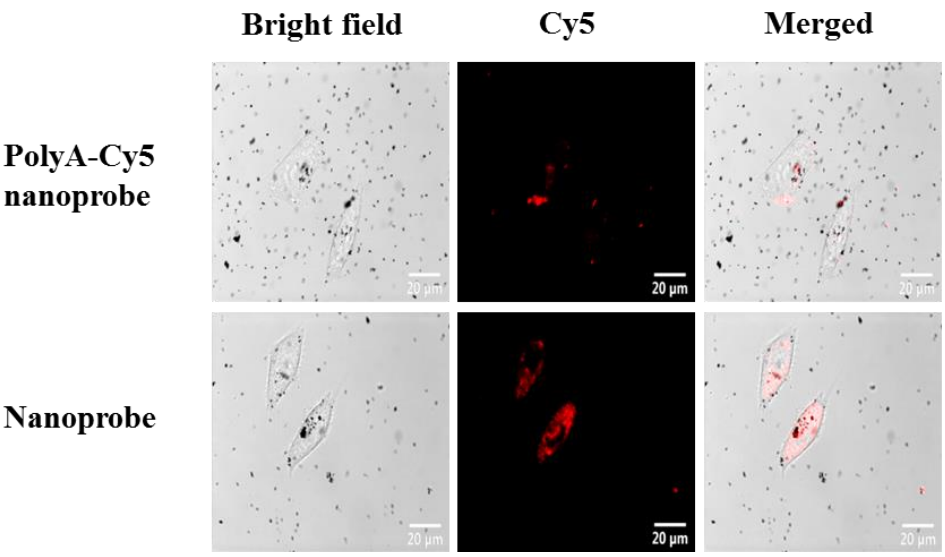

Figure S13 Fluorescent confocal micrographs of TK1 mRNA imaging with polyA$_{21}$-Cy5 nanoprobe and nanoprobe in HepG2 cells.

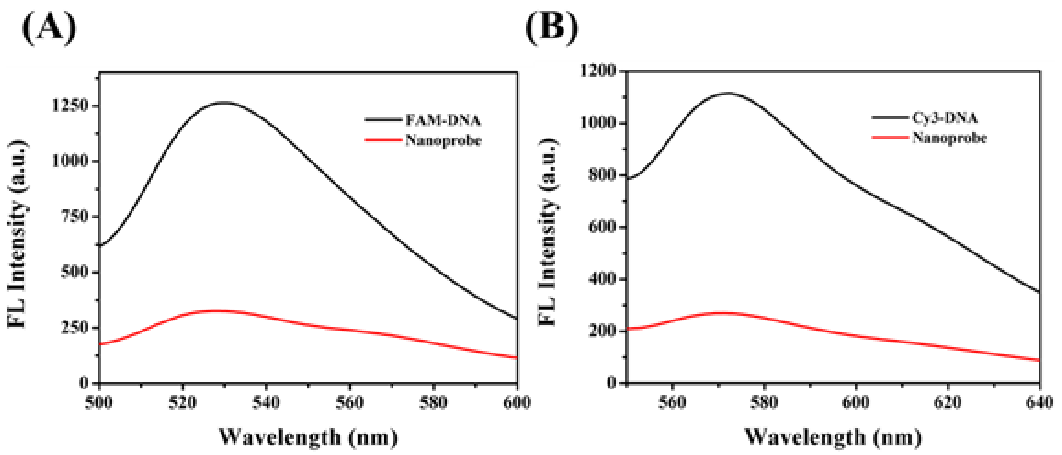

Figure S14 The changes of fluorescence intensity of the three dyes (FAM, Cy3 and Cy5) in the presence of Au@PDA NPs with different excitation wavelengths. (A) FAM labeled ssDNA (FAM-DNA) with 488 nm excitation wavelength. (B) Cy3 labeled ssDNA (Cy3-DNA）with 540 nm excitation wavelength.

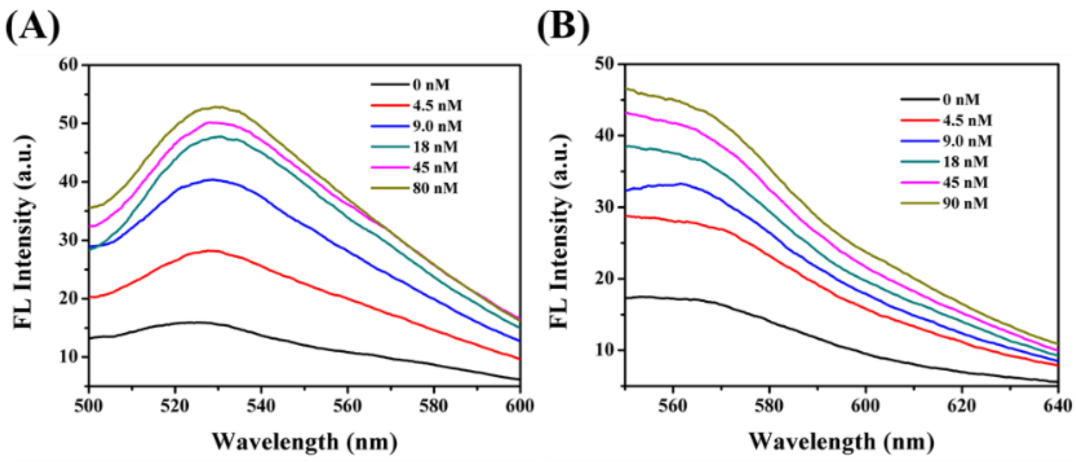

Figure S15 Representative fluorescence spectra for nanoprobe in the presence of various concentrations of mRNA targets measured with different excitation wavelength, respectively. (A) FAM labeled ssDNA (FAM-DNA) for C-myc mRNA detection with 488 nm excitation wavelength. (B) Cy3 labeled ssDNA (Cy3-DNA) for GalNAc-T mRNA detection with 540 nm excitation wavelength.

Table S4 DNA sequences used in the experiment.

Entry	Sequence (5′ - 3′)
Cy5-DNA	Cy5-GCG AGT GTC TTT GGC ATA CTT
TK1 mRNA	AAG TAT GCC AAA GAC ACT CGC
polyA-Cy5	Cy5-AAA AAA AAA AAA AAA AAA AAA
mis-1	AAG TAT GCC AAA GAC ACT CCC
mis-3	AAG TAT GCC AAA CAC AGT CCC
mis-5	AAC TAT GCG AAA CAC AGT CCC
miRNA-21	TAG CTT ATC AGA CTG ATG TTG A
FAM-DNA	FAM-AGG CTG CTG GTT TTC CAC TAC
C-myc mRNA	GTA GTG GAA AAC CAG CAG CCT
Cy3-DNA	Cy3-TCT TAT GCG GAT AGT GAA AGC
GalNAc-T mRNA	GCT TTC ACT ATC CGC ATA AGA
